# Smart Agriculture Development: How Can Rural Digital Transformation Enhance the Resilience of Food Security?

**DOI:** 10.3390/foods15030426

**Published:** 2026-01-24

**Authors:** Yingjie Song, Yi Song, Qiusu Wang

**Affiliations:** School of Finance, Shandong Technology and Business University, Yantai 264000, Chinasy230218tax@163.com (Y.S.)

**Keywords:** digital rural development, food security resilience, difference-in-differences method

## Abstract

The essential prerequisite for the state to ensure the stable production and supply of grain and other key agricultural products is to enhance food security resilience and transform traditional agricultural production and management models. This study utilizes panel data from major grain-producing counties in China from 2012 to 2023. Adopting the 2020 “National Digital Rural Pilot Program” as a quasi-natural experiment, it applies a difference-in-differences (DID) model to assess the program’s impact on food security resilience and its underlying mechanisms. The results demonstrate that digital rural development has a significant driving effect on food security resilience, with more pronounced effects observed in Southern regions, areas endowed with abundant labor resources, and regions with lower economic development levels. Mechanism analyses indicate that digital rural development plays a role in enhancing food security resilience through scaled grain operations and agricultural technological progress. Furthermore, resource allocation efficiency and fiscal transparency exert a positive regulatory effect in impacting food security resilience through digital rural development. This study elucidates the mechanism through which digital rural development enhances food security resilience, offering valuable policy insights for the coordinated advancement of rural revitalization and agricultural digitization.

## 1. Introduction

Food is the cornerstone of national security, food security is the fundamental guarantee for the stability and sustainable development of the national economy and society, and food security resilience serves as the critical support for withstanding risks and consolidating the baseline of food security. Together, these three components constitute the material foundation and security guarantee for national stability, social development, and public well-being. Currently, a confluence of multiple risk factors is synergistically exacerbating threats to global food security, including increasingly severe global climate change [1], a resurgence of geopolitical conflicts [2], and heightened volatility in international food trade [3]. Some low-income countries are even facing food shortages. Food security resilience, which denotes the capacity of food systems to withstand shocks and stresses while maintaining essential functions, constitutes a fundamental pillar for ensuring stable food production and sustainable supply. As the world’s most populous nation, China has been making sustained and substantive contributions to global food security and its resilience through a series of effective and systematic measures. During the 20th National Congress of the Communist Party of China, the government articulated the strategy to “comprehensively consolidate the foundation of food security”, delineating a systematic framework to contribute to food security resilience. Correspondingly, the 2025 Central Document No. 1 explicitly emphasizes the imperative to “continuously enhance the supply security capacity of grain and other key agricultural products”. By implementing targeted measures, including “stabilizing cultivated areas while enhancing yields, reinforcing technological support, and improving income safeguards”, it seeks to efficiently operationalize capacity-building programs that help bolster the resilience of the grain system to risks and ensure a stable supply.

Despite a sustained average annual growth rate of 1.45 percent in China’s grain output over the past decade, signifying long-term steady progress, significant disparities persist in grain production efficiency and cultivated land quality relative to developed nations. These gaps are further amplified by the international context, resulting in accumulating external pressures on China’s food security. Frequent extreme climate events, such as droughts, floods, and typhoons across various regions, have substantially disrupted the normal growth cycles of grain crops, impeded biomass accumulation, and consequently diminished yields. This situation underscores the structural vulnerabilities within China’s food security system when exposed to abrupt external environmental shocks [4]. Meanwhile, the reallocation of land away from grain production amid urbanization and agricultural modernization [5], combined with the low profitability of grain cultivation that discourages farmers’ planting enthusiasm, has created systemic incentive distortions [6]. These internal factors collectively undermine the endogenous stability of food security. This indicates that ensuring China’s food security extends beyond maintaining total grain output and necessitates a transition toward greater resilience.

In 2020, China officially launched the “National Digital Rural Pilot Program”, a strategic measure designed to contribute to the resilience of its food security system. Since its implementation, the digital rural pilot policy has contributed to agricultural modernization [7] by leveraging advanced technological innovations to increase grain yield and quality [8], improve land productivity [9], and augment human capital levels [10,11]. The adoption of digital agricultural technologies, particularly integrated water–fertilizer irrigation and precision farming systems, has increased grain yield per unit area, accounting for over 70 percent of the total growth in grain production. The application of digital technology and smart agriculture is associated with the advancement of traditional agricultural production through technological innovation [12]. By integrating agricultural production factors, it contributes to reducing grain production losses in quantitative terms [13] and enhancing resource allocation efficiency in qualitative terms [14]. This approach strengthens the stability and adaptability of the grain supply chain, thereby reinforcing the system’s resilience to external shocks and providing solid support for stable production capacity. Concurrently, as the digital rural initiative advances, over 90% of China’s administrative villages have achieved 5G coverage. Agricultural big data platforms, established through digital technologies, facilitate the real-time monitoring of national cropland cultivation status. This effectively curbs the conversion of farmland to non-agricultural and non-grain uses [15], thereby fortifying a solid defense for the stability of grain planting areas. Evidently, the implementation of digital rural development not only effectively mitigates external risks and internal structural contradictions within the grain industry but also constitutes a core pathway for enhancing food security resilience.

Since resilience thinking was introduced into agricultural research, food security resilience has been predominantly defined as the capacity of a food system to sustain its essential functions and structural integrity when confronted with diverse disturbances [16]. Existing research defines food security resilience as the ability of food systems to withstand shocks while preserving systemic stability [17]. The new era necessitates building a sustainable food security system that balances quantity, quality, and ecological sustainability, marking a strategic shift from a yield-centric paradigm toward multidimensional coordination [18]. Building upon this foundation, the core essence of food security resilience discussed in this study lies in the fundamental enhancement of a food system’s risk resistance capacity. This is achieved through structural adjustments and functional optimization in response to internal and external disturbances such as extreme weather and market volatility. Such resilience not only ensures stable food supply but also improves food quality, thereby realizing a “qualitative transformation”. It is not merely about immediate growth in quantitative indicators like food production volume or yield per unit area to achieve “quantitative expansion” [19]. Existing studies indicate that although the overall resilience of national food security has exhibited a fluctuating upward trajectory [20], significant regional disparities persist, with resilience levels being higher in central and eastern regions and lower in the western region, particularly more pronounced in major grain-producing areas [21]. Concurrently, the development of the current food system encounters a complex landscape shaped by multiple intertwined factors. Externally, uncertainties such as climate change [22,23], geopolitical conflicts [24], and economic cyclical fluctuations [25] continue to impose persistent impacts. Internally, key elements including the foundational conditions for agricultural production [26], the efficacy of scientific and technological innovation conversion [27], the implementation outcomes of policy subsidies [28,29], and the structure of the supply chain system still require substantial improvement [30]. Compounded by the inherent vulnerabilities of the grain industry and structural contradictions derived from imbalanced internal incentive mechanisms [31], this situation underscores the fact that enhancing resilience cannot be accomplished through internal self-adjustment and optimization alone. It urgently demands external support and systemic reforms to create synergistic effects. Digital technologies, encompassing big data, cloud computing, and artificial intelligence, are propelling a profound paradigm shift in agriculture. This transformation derives from the robust data analysis and processing capabilities, as well as intelligent decision-support functionalities, inherent in digital technologies [32]. It injects a revolutionary driving force into the systematic enhancement of food security resilience. Unlike traditional approaches that primarily focus on short-term productivity gains, digital rural development achieves intelligent cultivation models through the deep integration of digital technologies across the entire agricultural value chain. This enhances the risk response capabilities of agricultural producers, thereby providing systematic external support for strengthening food security resilience. At this critical juncture of agriculture’s transition towards digitalization, informatization, and intelligent transformation, major grain-producing regions carry the vital responsibility of safeguarding national food security [19]. Hence, advancing the deep integration of digital rural development with agricultural production has emerged as a pivotal strategic pathway, one that contributes to the overall competitiveness of agriculture and guarantees stable supply in major grain-producing areas [33].

To achieve this, this study concentrates on major grain-producing areas as its research focus. It establishes an evaluation framework for food security resilience grounded in three core dimensions: resistance capacity, recovery capacity, and transformation capacity [34]. Utilizing this framework, the study examines the impact of the “National Digital Rural Pilot Program” policy on food security resilience and investigates its underlying mechanisms. The primary contributions of this research are threefold: (1) Perspective innovation—digital rural development represents a crucial policy instrument for promoting agricultural modernization. However, its potential effect on food security resilience has not yet undergone systematic empirical assessment. This study offers a novel perspective for utilizing digital rural initiatives to enable resilience governance and ensure food security. (2) Methodological advancement—methodologically, this study diverges from prior approaches that often relied on subjective comprehensive evaluation methods to measure digital rural development levels. By conceptualizing the “National Digital Rural Pilot Program” as a quasi-natural experiment and applying the difference-in-differences method, it mitigates the subjectivity inherent in evaluating digital rural initiatives, thereby furnishing more objective empirical evidence for national digital rural development. (3) Content enrichment—regarding research content, this study delves into the actual transmission mechanisms of digital rural development policies and elucidates their pathways for enhancing food security resilience. Specifically, from the standpoints of large-scale grain operations and agricultural technological advancement, it clarifies the mechanism through which digital rural development contributes to food security resilience, separately verifying the mediating roles of large-scale operations and agricultural technological progress. Furthermore, considering issues such as information asymmetry and the urban–rural digital divide, it investigates the moderating effects of resource allocation efficiency and fiscal transparency within the process of digital rural development enhancing food security resilience.

## 2. Theoretical Analysis

**Figure 1:**
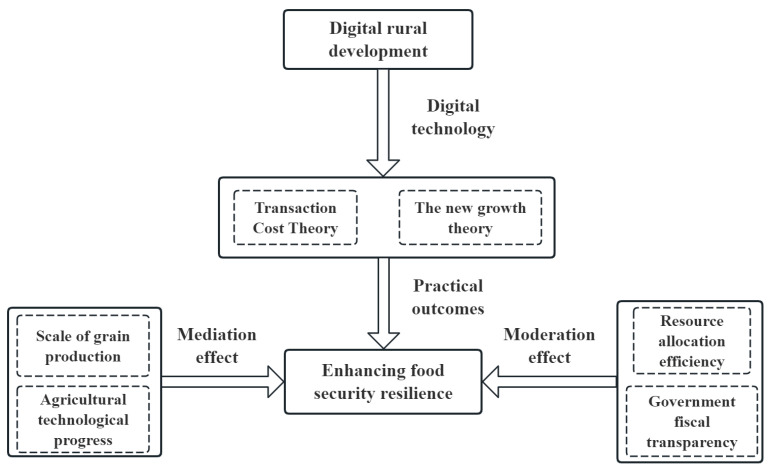
Theoretical analysis framework.

The essence of digital rural development lies in utilizing digital technology as the core driving force to bridge the urban–rural digital divide, thereby promoting the transformation of rural areas from traditional models towards digitalization, intelligence, and modernization. This approach addresses fundamental challenges in rural development, such as information asymmetry and inefficient resource allocation, and plays a critical role in enhancing food security resilience. The theoretical analytical framework illustrating the impact mechanism of digital rural development on food security resilience is presented in Figure 1.

### 2.1. The Direct Impact of Digital Rural Development on Food Security Resilience

From the perspective of new growth theory, technological progress constitutes the core driver of economic growth. In contrast to the “extensive growth” model driven by disordered resource inputs, the decisive force propelling sustainable economic development resides in achieving optimal resource allocation, transforming production methods, and enhancing productivity through endogenous technological progress [35]. Digital rural development leverages technologies such as the Internet, big data, cloud computing, and artificial intelligence to foster the deep integration of digital technologies with agricultural production practices, thereby providing comprehensive technological support for food security. By utilizing big data and information technology, agricultural resource inputs can be precisely identified and controlled, resulting in a reduction in the application of agricultural chemicals, including pesticides, plastic mulch, and fertilizers [36]. This approach not only optimizes grain production models and diminishes reliance on resource endowments but also enhances grain production efficiency and quality, consequently contributing to the foundation for sustainable food security. Meanwhile, grounded in transaction cost theory, digital rural development systematically reduces transaction costs throughout the entire grain supply chain. This addresses the pain points inherent in traditional grain systems, such as information asymmetry, opaque processes, and inadequate oversight, directly mitigating market friction and information gaps [37]. Consequently, it enhances the grain system’s operational efficiency and flexibility in risk response, bolsters the grain industry chain’s capacity to buffer against market fluctuations, and consolidates its overall resilience in risk management [38]. Based on the foregoing analysis, the first research hypothesis is proposed.

**Hypothesis** **1.** *Digital rural development is conducive to ensuring food security resilience.*

### 2.2. The Indirect Impact of Digital Rural Development on Food Security Resilience

(1) The mediating effect of scale operations in grain production and agricultural technological progress:

Large-scale grain operations represent a pivotal pathway for modern agricultural development, signifying the transformation and upgrading of traditional smallholder farming models towards modernized and intensive practices. Grounded in the theory of economies of scale, replacing fragmented farming with centralized and standardized management can effectively address the inherent issues of low production efficiency and high production costs in traditional smallholder agriculture. Digital rural development vigorously propels the advancement of large-scale grain operations by enhancing agricultural productivity, establishing integrated agricultural industrial systems, and advancing agricultural infrastructure projects, thereby facilitating scaled and efficient operational management. Furthermore, digital rural initiatives stimulate the growth of non-agricultural industries. Digital platforms, exemplified by rural e-commerce and live streaming, provide farmers with diverse employment opportunities [39], facilitate the orderly transfer of rural labor, and create favorable conditions for farmland consolidation. This lays a practical foundation for farmers to expand their operational land [40]. Large-scale grain operations contribute to reducing the application rates of fertilizers and pesticides [41], while enhancing the operational efficiency of smart irrigation systems can boost grain yield per unit area [42]. By enabling grain producers to achieve relatively stable economic returns, large-scale operations effectively enhance their enthusiasm for grain cultivation. This approach helps prevent frequent adjustments to cropping structures, ensures sustained and stable grain yields, and strengthens the adaptability and resilience of grain production against risks. Based on this analysis, the second research hypothesis is proposed.

**Hypothesis** **2.** *Digital rural development may impact food security resilience by expanding the scale of grain production operations.*

Agricultural technological progress serves as a pivotal driver of modern agricultural development, representing the transformation and upgrading of traditional agricultural models towards modern intensive and intelligent approaches. Grounded in factor substitution theory, the replacement of labor with machinery can effectively mitigate the dual challenges of agricultural labor shortages and suboptimal labor quality. Digital rural development establishes a robust support system for agricultural technological advancement through comprehensive data empowerment [43]. Specifically, the integration of diverse datasets, such as agricultural meteorological information, soil moisture conditions, and crop growth cycles, through large-scale data platforms accelerates the research and development of key technologies like biological breeding. This process facilitates the cultivation of grain varieties with inherent stress tolerance [44]. Concurrently, digital channels, exemplified by rural e-commerce and agricultural technology applications, can be utilized to promote the large-scale application of precision planting and intelligent monitoring technologies in grain production [45]. Furthermore, advancements in agricultural technology help alleviate structural labor shortages, particularly during critical farming seasons like planting and harvesting. This enhancement boosts labor productivity and agricultural output [46], ensuring the continuity and stability of food production. More significantly, progress in agricultural technology fosters the deep integration of scientific and technological innovation with digital agriculture, accelerates agricultural modernization, contributes to the resilience of agricultural systems against natural disasters, and improves their adaptability to climate change, thereby laying a solid foundation for the sustained effect of food security resilience [47]. Based on this analysis, the third research hypothesis is proposed.

**Hypothesis** **3.** *Digital rural development may impact food security resilience through agricultural technological advancement.*

(2) The Moderating Effects of Resource Allocation Efficiency and Fiscal Transparency

Digital rural development’s role in enhancing food security resilience cannot rely solely on direct technological investment. Its effectiveness depends fundamentally on the rational flow and efficient utilization of resources within agricultural production systems. In this process, resource allocation efficiency acts as the critical link connecting technological inputs to the realization of their potential outcomes. By optimizing the flow and application of information within digital rural initiatives, improved resource allocation guides agricultural production factors, such as land and labor, toward more efficient sectors. This fosters a better match between agricultural production and market demand, reduces resource waste caused by factor misallocation [48,49], and enhances the flexibility of the food production system in responding to risks and disruptions. Furthermore, enhancing resource allocation efficiency contributes to the integration of digital technologies with agricultural production, which accelerates the dissemination and application of advanced agricultural technologies. Directing resources like capital and talent toward agricultural R&D and implementation alleviates resource constraints and elevates the technological resilience of grain production against various risks [50]. Finally, resource allocation efficiency helps drive coordinated integration across the digital rural industrial chain. It optimizes resource distribution throughout the entire grain supply chain [51], eliminates bottlenecks, enhances the stability and adaptability of the grain supply system, and contributes to its capacity to withstand sudden shocks, thereby comprehensively increasing food security resilience. Based on this analysis, the fourth research hypothesis is proposed.

**Hypothesis** **4.** *Resource allocation efficiency exerts a positive moderating effect on the resilience of food security in the context of digital rural development.*

Fiscal transparency serves as a critical safeguarding mechanism for enhancing the effectiveness of digital rural development and contributing to food security resilience. By standardizing fund use processes, guiding multi-stakeholder participation, and optimizing rural governance systems, these measures provide essential support for enabling digital rural development to bolster food security resilience. First, fiscal transparency improves the transparency and traceability of fund usage in digital rural initiatives [52], effectively reducing misallocation and inefficiency in projects such as the digital transformation of agricultural infrastructure and grain production monitoring systems. This ensures resources are directed toward critical components of food security safeguards, thereby contributing to the material foundation of the grain production system’s resilience against risks. Second, fiscal transparency helps enhance regulatory compliance in digital rural policies [53]. By mitigating risks associated with social capital participation in digital infrastructure development related to food security, it attracts more funding toward the digital upgrading of grain production, alleviates financial pressures, and ultimately enhances the risk resilience of the grain industry chain. Finally, fiscal transparency strengthens public oversight and participation mechanisms. Through the disclosure of public fund allocation and use, the information disclosure mechanism of digital government motivates farmers, cooperatives, and other stakeholders to engage in the digital transformation of grain production. This fosters a collective societal effort to safeguard food security, thereby achieving greater resilience in food security. Based on this analysis, the fifth research hypothesis is proposed.

**Hypothesis** **5.** *Fiscal transparency exerts a positive moderating effect on the resilience of food security in the context of digital rural development.*

## 3. Methodology

### 3.1. Variable Setting

#### 3.1.1. Dependent Variable

Food security resilience (FSR) is defined as a food system’s capacity to maintain or rapidly restore stable supply and ensure food quality and safety during shocks. This capacity also includes adaptive and transformative capabilities to continuously meet nutritional needs. Specifically, FSR encompasses three core dimensions: risk resistance, crisis recovery, and transformative capacity [19,54]. Risk resistance refers to a system’s ability to mitigate damage and maintain essential functions when confronting adverse factors like natural disasters and market fluctuations. A crisis recovery capability denotes the capacity of food systems to adapt swiftly to environmental changes and evolving social demands following shocks, thereby sustaining the provision of appropriate food products and services. Transformative capacity indicates a food system’s capability to undergo fundamental reforms and innovations in response to long-term challenges and structural issues, achieving transformation and upgrading to meet new development requirements. Guided by principles of scientificity, comprehensiveness, and data availability, an indicator system is structured into three levels: 3 primary, 6 secondary, and 12 tertiary indicators (Table 1).

It is worth noting that, in the indicator attributes, positive indicators represent the promotion of food security resilience. For example, the larger the “cultivated land area”, the more stable the foundation for agricultural production and the stronger the resilience to risks. The higher the “grain yield per unit area”, the better the robustness of production and supply. Chemical Fertilizer Application is a negative indicator; the higher the “fertilizer application rate per unit area”, the greater the potential for soil pollution and ecological pressure, which may reduce the sustainability of agriculture. These indicators are determined using an entropy method for model fitting.

#### 3.1.2. Independent Variable

The independent variable in this study is the policy variable ${Policy}_{it}$, indicating whether a county i in China’s major grain-producing regions implemented the digital rural development policy in t. This variable is constructed as a binary indicator; if a county or district is designated as a digital rural pilot area in year t, the policy variable takes a value of 1 from that year onward. Otherwise, it remains 0.

#### 3.1.3. Mechanism Variables

(1) Mediating variables

The scale of grain production (SGP) is quantified as the ratio of cultivated land area to the number of individuals employed in agriculture, forestry, animal husbandry, and fisheries. This measure reflects the degree of concentration and economies of scale in grain production within the agricultural sector, characterizing the scale attributes resulting from resource aggregation directed toward grain production. A prudent expansion of scale facilitates the realization of intensive advantages in machinery and technology, thereby contributing to food security resilience.

Agricultural technological progress (ATP) is measured by the ratio of agricultural machinery power to cultivated land area, indicating the level of technological innovation, application, and upgrading in agricultural production. The penetration of advanced technologies into all stages of agricultural production provides solid technical support for enhancing food security resilience by improving production efficiency and increasing yield per unit area.

(2) Moderating Variables

Resource allocation efficiency (RAE) is quantified as the ratio of total grain output to cultivated land area. This measure reflects the importance of grain production within the agricultural sector and the effectiveness of resource allocation.

Government fiscal transparency (GFT) reflects the extent to which governmental bodies disclose information regarding fiscal investments and the implementation of food-security-related policies. By promoting the rational allocation of public funds and enhancing policy predictability, such transparency helps build a solid fiscal foundation to consolidate food security resilience.

#### 3.1.4. Control Variables

Based on established research [55], this study selects the following variables as controls:

Labor resources (LRs), expressed as the proportion of persons employed in agriculture, forestry, animal husbandry, and fisheries relative to the total rural workforce. Agricultural Industrial Structure (AIS), measured by the ratio of the gross output value of agriculture to the county’s gross domestic product. Economic development level (EDL), represented by the per capita GDP. Level of Agricultural Mechanization (LAM), quantified as the ratio of the mechanically harvested area to the total cultivated land area. Transportation Accessibility (TA), measured by the ratio of the total highway mileage to the size of the administrative area. The non-agricultural economic share (NAE) is measured by the ratio of the added value of the secondary and tertiary industries to the total regional output value.

### 3.2. Data Sources

This study employs panel data from 1500 counties in major grain-producing regions covering the period 2012–2023 to examine how the National Digital Rural Pilot Policy enhances food security resilience. The list of pilot areas is obtained from the “National Digital Rural Pilot Regions List” for major grain-producing regions, jointly issued in 2020 by seven government entities, including the Cyberspace Administration of China. Urban districts and counties with significant data deficiencies are excluded, resulting in a final sample of 1198 counties. Data are sourced from the China Statistical Yearbook, China Rural Statistical Yearbook, China County Statistical Yearbook, China Fiscal Transparency Report, and statistical bulletins on national economic and social development published by individual counties. For variables with limited missing observations, linear interpolation is used to fill gaps. To reduce potential issues such as heteroscedasticity, all continuous variables included in the empirical analysis are transformed using natural logarithms.

Furthermore, based on the methodological approach of Colin Cameron et al. [56], this study clusters standard errors at the county level. This approach is necessary because data organized at the city or county level often exhibit correlated error terms within the same county, due to shared economic, political, cultural, and environmental influences. Applying county-level cluster-robust standard errors to Equations (1)–(3) corrects for this within-county correlation and ensures the robustness of the baseline regression results. This practice is grounded in existing research and addresses the potential bias that could arise from ignoring such clustering.

Table 2 presents the descriptive statistics for key variables. The mean of the dependent variable (FSR) is 2.2463 with a standard deviation of 0.4078, suggesting moderate variability around the mean. The minimum value of 0.9700 and maximum value of 4.9852 show significant variation, reflecting substantial differences across samples. Firstly, the attributes of indicators differ. The indicator system includes both positive and negative indicators. For instance, the positive indicator “cultivated land area” and the negative indicator “fertilizer application rate per unit area” can amplify the range span when extreme values occur. Secondly, the sample regions exhibit inherent heterogeneity in resource endowments and agricultural production scale. For instance, Jiangsu Province demonstrates a higher food security resilience index than Hebei Province. Leveraging superior natural endowments, well-developed agricultural infrastructure, and large-scale intensive production, Jiangsu achieves significantly greater food security resilience than Hebei. Conversely, Hebei faces constraints due to uneven precipitation, suboptimal soil quality, insufficient investment in agricultural infrastructure, and fragmented smallholder farming operations, resulting in weaker stability in grain production. Finally, different regions face varying intensities of external shocks such as natural disasters and market fluctuations, and their risk response systems differ in sophistication. These factors collectively influence variations in the food security resilience index.

### 3.3. Baseline Model Specification

In 2020, the Cyberspace Administration of China issued the “Notice on Launching the National Digital Rural Pilot Program”, designating 117 counties (cities and districts) as the first batch of national level digital rural pilot areas. This initiative marked the official launch of the digital rural pilot phase. The policy aims to bridge the urban–rural digital divide through information technology, promote the growth of the rural digital economy, and support the intelligent transformation of agricultural production alongside the digitization of agricultural operations.

Accordingly, this study treats the implementation of the “National Digital Rural Pilot Program” as a quasi-natural experiment. It employs a difference-in-differences model (DID) to examine the impact of the digital rural development policy on food security resilience in major grain-producing regions. The benchmark model is specified in Equation (1) below:

(1) $$Y_{it}\;=\;{\;\beta }_{0}+\beta_{1}{Policy}_{it}+\beta_{2}X_{it}+\mu_{i}+\theta_{t}+\varepsilon_{it}$$

In the model, $Y_{it}$ is the dependent variable, denoting food security resilience in major grain-producing regions, i indexes the county, and t denotes the year. The independent variable ${Policy}_{it}$ represents the “Digital rural development” policy variable and is defined as the interaction term ${Policy}_{it}\;$ = ${Treat}_{i}*{Post}_{t}$. ${Treat}_{i}$ is the treatment variable, assigned a value of 1 for counties implementing digital rural development during the sample period, assigned 0 otherwise; ${Post}_{t}$ represents a policy timing indicator, taking a value of 1 in the year a county implements digital rural development and 0 otherwise. The policy variable ${Policy}_{it}$ is a dummy variable that indicates whether county i is designated as a national digital rural pilot zone in year t (1 if designated, 0 otherwise). $X_{it}$ represents a set of control variables. The model incorporates county-fixed effects $\left(\mu_{i}\right),$ year-fixed effects $\theta_{t}$, and a random disturbance term $\varepsilon_{it}$. $\beta_{0}$ is the constant term, $\beta_{2}$ denotes the estimated coefficient for control variables, and the coefficient $\beta_{1}$ measures the impact of digital rural development policies on food security resilience in pilot counties within major grain-producing regions, which is the primary focus of this study.

### 3.4. Mediation Effect Model Specification

To empirically investigate the pathway through which the implementation of digital rural development policies enhances food security resilience, a two-step mediation analysis is conducted based on Equation (1). The model is constructed as follows in Equation (2) below:

(2) $$M_{it}\;=\;\tau_{0}+\tau_{1}{Policy}_{it}+\tau_{2}X_{it}+\mu_{i}+\theta_{t}+\varepsilon_{it}$$

In the model, $M_{it}$ denotes the mediating variable, which is expressed in logarithmic form. The mediating variables comprise the scale of grain production (SGP) and agricultural technological progress (ATP). Here, $\tau_{1}$ represents the estimated coefficient of digital rural development policy on the mediating variables, namely agricultural technological progress and grain-scale operations. All other symbols are consistent with those defined in Equation (1).

### 3.5. Moderation Effect Model Specification

To examine the moderating roles of resource allocation efficiency (RAE) and government fiscal transparency (GFT), an interaction term involving these variables is introduced into Equation (1), yielding the moderation effect model specified in Equation (3) below:

(3) $$Y_{it}\;=\;\eta_{0}+\eta_{1}{Policy}_{it}+\eta_{2}N_{it}+\eta_{3}N_{it}*{Policy}_{it}+\eta_{4}X_{it}+\mu_{i}+\theta_{t}+\varepsilon_{it}$$

In the model, $N_{it}$ represents the moderating variables, which include RAE and GFT. The original data for these moderators are standardized to mitigate potential multicollinearity concerns. The analysis focuses primarily on the coefficient $\eta_{3}$.

## 4. Analysis and Results

### 4.1. Analysis of Baseline Model Results

Table 3 presents the estimated impact of digital rural development on food security resilience. Column (1) reports a coefficient of 0.1440 without incorporating control variables or county and time-fixed effects, which is statistically significant at the 1% level, indicating a preliminary positive association. After including county and time fixed effects in Column (2), the estimated coefficient decreases to 0.0876 remains significant, confirming the robustness of the relationship to basic unobserved heterogeneity. In Column (3), control variables are introduced to the specification in Column (1) to mitigate potential omitted variable bias, yielding a coefficient of 0.1330, significant at the 1% level. Column (4) incorporates both county-level and time fixed effects into the model from Column (3), resulting in a coefficient of 0.0898 that remains significant. This indicates that following the implementation of digital rural development policies, food security resilience increased by an average of approximately 8.98%**.** Overall, the consistently positive and significant coefficients across specifications provide robust evidence in support of Hypothesis 1. This finding indicates that pilot counties can further demonstrate the effectiveness of digital rural development when addressing risks such as extreme weather events, pest and disease outbreaks, and fluctuations in agricultural input prices. Specifically, in 2024, China’s total grain output attained a historic milestone of 700 million tons, while the per capita grain possession sustained a level above 500 kg, substantially surpassing the internationally recognized food security threshold. China’s total grain output has achieved long-term steady growth. The implementation of the Digital Village Initiative has provided crucial practical experience for rural digital transformation and high-quality agricultural development.

### 4.2. Parallel Trend Analysis

**Figure 2:**
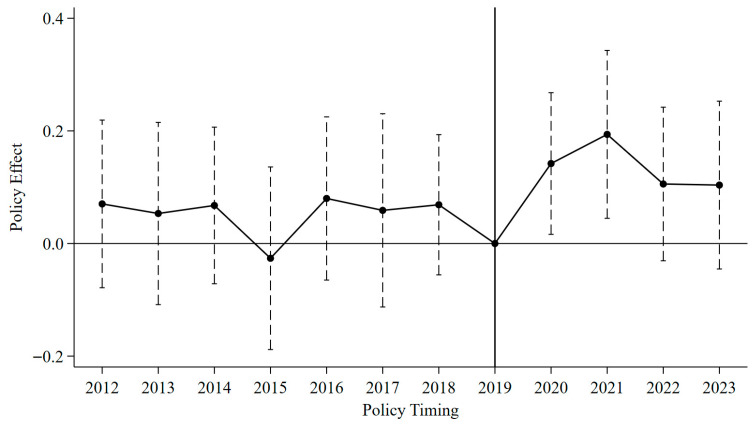
Parallel trend test.

In empirical analyses utilizing the DID approach, the parallel trends assumption is a critical prerequisite for obtaining unbiased causal estimates. This assumption ensures that observed changes in outcomes following a policy intervention are not driven by pre-existing differential trends between the treatment and control groups. To examine whether significant differences in food security resilience existed between pilot and non-pilot regions prior to the implementation of digital rural development policies, this study employs an event study framework to test the validity of the parallel trends assumption—a fundamental requirement for the DID model. Figure 2 displays the estimated coefficients along with their 90% confidence intervals for the periods preceding the policy intervention. Reassuringly, all estimated coefficients prior to the policy implementation are insignificant and economically close to zero. This pattern supports the validity of the parallel trends assumption and justifies the application of the DID framework for causal inference.

It is noteworthy that in 2022 and 2023, the policy effect estimate declined in 2023 but remained positive. This attenuation may be influenced by external factors, such as extreme weather events and fluctuations in the international grain market, which could have undermined the policy’s effectiveness. Concurrently, the law of diminishing marginal returns in digital government policies has become evident at this stage, as the release of benefits in the early stage stabilizes and future growth potential becomes limited. Consequently, the interplay of these factors results in the observed phased temporal characteristics of the policy effects.

### 4.3. Robustness Tests

#### 4.3.1. Temporal Placebo Test

This study conducts a temporal placebo test by altering the assumed policy implementation timing to isolate the influence of temporal-specific variations on food security resilience between the treatment and control groups. Adopting the counterfactual framework [57], we assume that the policy was implemented one, two, three, and four years prior to the actual date to construct counterfactual policy timelines, and subsequently re-estimate Equation (1). As reported in columns (1) to (4) of Table 4, the estimated coefficients for the placebo policy indicator are insignificant, which helps eliminate confounding effects from non-policy factors and verifies that the temporal placebo test is satisfied. This result affirms that the observed enhancement in food security resilience is attributable to the digital rural development policy. Furthermore, no significant differences in food security resilience were observed between pilot and non-pilot counties during the pre-policy periods.

#### 4.3.2. Placebo Test for Fictional Counties

**Figure 3:**
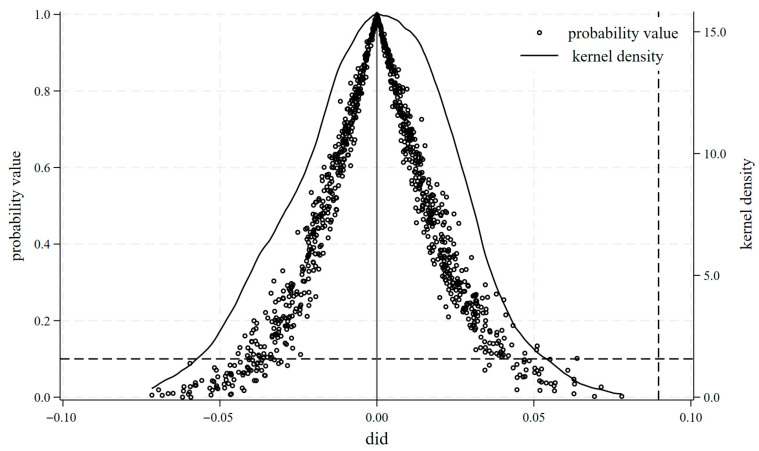
Placebo test in fictional counties.

To eliminate potential confounding effects from unobservable factors in assessing the impact of the digital rural development policy on food security resilience, this study implements a permutation-based placebo test for treatment group assignment, following the approach of Ferrara et al., to mitigate biases arising from random or unobserved events and to enhance the robustness of the benchmark regression results [58]. Specifically, a placebo policy indicator is constructed through 500 random sampling iterations and incorporated into Equation (1) for estimation. Figure 3 presents the estimated coefficients of these placebo policy variables and their distributions of statistical significance. The results show that the regression coefficients for the placebo policy variable on food security resilience follow a normal distribution centered near zero. Most *p*-values associated with the placebo coefficients exceed 0.1, failing to attain the 10% significance level, and are substantially distant from the actual estimated coefficient. Comparisons reveal significant differences in the benchmark results, 0.0898, indicating that the effect of the digital rural pilot policy on food security resilience is statistically robust. After excluding the influence of random factors through testing, the reliability of the research conclusions is further affirmed.

#### 4.3.3. Propensity Score Matching with Difference-in-Differences (PSM-DID) and Entropy Balance Test Results

To mitigate potential selection bias in designating “National Digital Rural Pilot Counties”, which could introduce endogeneity into the benchmark regression estimates, this study employs a PSM-DID approach, supplemented by an entropy balancing test [59,60]. This combined strategy aims to reduce sample selection bias and assess the robustness of the baseline findings. As shown in Column (2) of Table 5, the coefficient for ${Policy}_{it}$ is 0.0896, which is significant and comparable to the baseline estimate. Column (3) reports a coefficient of 0.0838 for ${Policy}_{it}$, which remains positive and significant. These findings demonstrate that, even after entropy balancing adjustment, digital rural development maintains a positive and significant impact on food security resilience in pilot regions. Thus, after accounting for potential selection bias and other unobserved confounders, digital rural development is found to enhance food security resilience, and the benchmark regression results are robust.

#### 4.3.4. Eliminate Interference from Parallel Policies

During the sample period, food security resilience may have been affected by the implementation of other concurrent policies, which could challenge the robustness of the benchmark regression results. To isolate the net effect of the digital rural pilot policy and mitigate potential confounding from overlapping initiatives, this study excludes counties that were designated during the sample period as “National Modern Agricultural Demonstration Zones”, “National Safe Agricultural Machinery Demonstration Counties”, “National Pilot Units for Agricultural Product Quality and Safety Counties”, or “National Rural Revitalization Demonstration Counties”. Regression results reported in Columns (1) to (4) of Table 6 show that, even after accounting for potential interference from other contemporaneous policies, the digital rural pilot policy continues to exhibit a significant and positive effect on food security resilience. This outcome further validates the robustness of the benchmark findings.

### 4.4. Other Robustness Tests

#### 4.4.1. Year–Province-Fixed Effects Model

Provinces exhibit substantial heterogeneity in natural conditions, resource endowments, economic foundations, and policy preferences, which may systematically affect the dependent variable. To mitigate potential biases arising from these differences, the study incorporates year–province-fixed effects into the empirical model. This specification absorbs time invariant provincial characteristics and year-specific shocks common to all provinces, thereby isolating variation attributable to the policy intervention and yielding a more precise treatment effect estimate. Column (1) of Table 7 reports the results from estimating Equation (1) with year–province-fixed effects. The coefficient on the policy variable is 0.0835, significant at conventional levels, indicating a positive association between the digital rural development policy and food security resilience after controlling for time-varying province-specific unobservable factors and common temporal shocks. The consistency of this finding across alternative model specifications reinforces that the result is not spurious but reflects a robust causal relationship during the sample period.

#### 4.4.2. Exclusion of County-Level Cities

Although county-level cities and ordinary counties belong to the same administrative tier in China, they differ significantly in governmental authority and regional development strategies due to their distinct institutional settings. To mitigate potential confounding effects arising from these institutional disparities on the benchmark regression estimates, this study excludes county-level cities from the sample and re-estimates the model based on Equation (1). The regression results presented in Column (2) of Table 7 indicate that, after the exclusion of county-level cities, the estimated coefficient for the policy variable remains significant and positive, with a value of 0.1128. This finding further validates the robustness of the benchmark regression results.

#### 4.4.3. One-Period Lag of Policy

To account for potential time lags in the implementation of the digital rural pilot policy, which was officially initiated around 2021, a one-period lag of the policy variable is included in the regression analysis. The estimation results reported in Column (3) of Table 7 indicate that the coefficient for the lagged policy variable is 0.0295 and remains significant and positive. This result further confirms the robustness of the benchmark findings.

### 4.5. Analysis of the Mechanism by Which Digital Rural Development Affects Food Security Resilience

While this study provides a rigorous theoretical examination of the transmission mechanisms, empirical analysis using Equations (2) and (3) remains imperative to test the robustness of the theoretical predictions and the models’ applicability.

#### 4.5.1. Mediation Effect Test

Column (2) of Table 8 shows that the coefficient for the impact of digital rural development on the scale of grain production operations is 0.2461, which is significant. This indicates that pilot counties exhibit significantly higher levels of grain scale operations compared to non-pilot counties. Thus, digital rural development can enhance food security resilience by promoting grain scale operations. The deep integration of digital technology with agriculture, rural areas, and farmers has given rise to information platforms such as e-commerce platforms and farmland transfer platforms. These platforms facilitate farmers’ transition to non-agricultural employment, enhance the efficiency of agricultural land transfers, and promote large-scale farmland management [61]. Large-scale grain production, in turn, drives the adoption of green technologies and increases mechanization levels, further contributing to food security resilience [62]. Therefore, Hypothesis 2 is validated.

Furthermore, as shown in column (3) of Table 8, the coefficient value for digital rural development’s impact on agricultural technological progress is 0.2532 and is significant. It indicates that digital rural development significantly promotes agricultural technological progress, thereby contributing to food security resilience in pilot regions. The advancement of digital rural development has enhanced the rural technology R&D and extension system and has accelerated the flow and application of advanced technologies between urban and rural areas. This process creates opportunities for the upgrading and efficient transformation of agricultural technologies [43]. Simultaneously, promoting rural industrial integration and business model innovation extends the grain industry chain, enhances production quality, efficiency, and risk resilience [47]. Therefore, Hypothesis 3 is validated.

#### 4.5.2. Mediation Effect Test Based on the Bootstrap Method

Using the Bootstrap method, we further validated the mediating effect between digital rural development and food security resilience. To test for mediation effects using the Bootstrap method, we observed whether the confidence interval included zero. If it did not include zero, the mediation effect was significant; otherwise, it was not significant. The results in Table 9 indicate that the confidence intervals for the intermediary effect of digital rural development on promoting large-scale grain operations, calculated using the Bootstrap method, are [0.000014, 0.00025] and [0.00003, 0.00030], respectively. Both intervals significantly exclude zero. The mediation effect value is 0.00011. Similarly, the confidence intervals for the mediating effects of agricultural technological progress also significantly exclude zero; the mediation effect value is 0.00034. Furthermore, the fact that both the upper and lower bounds of these confidence intervals exceed zero indicates that the Bootstrap method validates the effectiveness of the mediating effect model proposed in this study, confirming the robustness of the results.

#### 4.5.3. Moderation Effect Test

Column (2) of Table 10 reports the regression results examining the moderating effect of resource allocation efficiency. The findings indicate that the coefficient value for the interaction term RAE* on food security resilience is 0.0722 and significant. This demonstrates that RAE exerts a positive moderating effect in the process of enhancing food security resilience in pilot regions through digital rural development. This can be attributed to the fact that efficient resource allocation facilitates the targeted deployment of advanced digital agricultural technologies, specialized talent, and capital within digital rural initiatives [50]. These resources can be allocated rationally to grain production processes to improve yield and quality, optimize regional grain distribution to mitigate supply shortage risks, and reduce production and circulation costs to stabilize grain prices. Simultaneously, it promotes the deep integration of digital rural development with the grain industry, thereby contributing to systemic risk resilience. Hypothesis 4 is thus supported.

Column (3) in Table 10 presents the regression results for the moderating effect of fiscal transparency. The estimated coefficient for the interaction term GFT* ${Policy}_{it}$ on food security resilience is 0.0716 and significant. This proves that GFT positively moderates the enhancement of food security resilience in pilot regions through digital rural development. This is primarily because fiscal transparency, through information disclosure and enhanced oversight mechanisms, facilitates improvements in the efficiency of fund utilization. It ensures that financial resources for digital rural development are accurately directed toward critical areas such as grain production and distribution, thereby reducing fund misappropriation and waste. This guarantees the effective implementation of digital projects, such as smart irrigation and grain traceability systems, ultimately elevating the level of digitalization in grain production and allocation [52]. Hypothesis 5 is validated.

### 4.6. Heterogeneity Analysis

#### 4.6.1. Regional Heterogeneity Analysis

Geographic spatial heterogeneity typically manifests as significant disparities in resource endowments, human capital accumulation, agricultural technological levels, industrial support systems, and infrastructure development. These variations systematically influence the efficacy of digital rural development policies in enhancing food security resilience. This study categorizes the 13 major grain-producing provinces into two broad zones: the Northern zone (including Shandong, Henan, Hebei, Inner Mongolia, Heilongjiang, Jilin, and Liaoning) and the Southern zone (comprising Anhui, Hubei, Hunan, Jiangsu, Jiangxi, and Sichuan), analyzing the differential impacts of digital rural development on food security resilience across these distinct geographical regions.

As presented in columns (1) and (2) of Table 11, the estimated coefficient for the impact of digital rural development on food security resilience in the Southern grain-producing regions is 0.2010, which is significant and positive. This indicates that following the implementation of digital rural development policies, the food security resilience of major grain-producing regions in the Southern regions increased by an average of approximately 20.10%. Conversely, in the Northern regions, the impact of digital rural development did not achieve statistical significance. The digital rural development in Southern China’s major grain-producing regions demonstrates more pronounced empowerment effects. This stems from three primary factors: First, limited per capita arable land and fragmented farming operations drive households to leverage digital technologies to overcome constraints, with these technologies highly compatible with specialty agriculture, precisely enhancing yield per unit area and resource efficiency. Second, the early deployment and extensive coverage of digital infrastructure grant these regions a head start in agricultural digitization, informatization, and intelligent development. Third, the development of non-agricultural industries has driven labor migration. The remaining farmers compensate for labor shortages through digital tools like drone crop protection. Long-term market exposure has also enhanced their digital literacy, making their contribution to food security resilience more pronounced [14]. Northern grain-producing regions face multiple constraints. Despite contiguous land parcels, large-scale cultivation relies on traditional machinery and experience. Digital transformation requires overcoming higher cost barriers such as land consolidation and agricultural equipment compatibility, resulting in slower technology adoption compared to that in Southern regions. Furthermore, farmers’ limited education and non-agricultural employment opportunities lead to low digital literacy, weakening their ability to adopt complex systems like agricultural big data. This further exacerbates regional disparities in digital transformation. Therefore, the Northern regions are at a disadvantage in digital infrastructure development and agricultural digital transformation, while the Southern regions are more likely to demonstrate tangible results.

#### 4.6.2. Analysis of Heterogeneity in Agricultural Labor Force Endowments

Agricultural labor endowment is a crucial factor in food production, and regional disparities in labor resources across regions can impact the effectiveness of digital rural development initiatives. This study categorizes the sample into two groups, namely high labor endowment and low labor endowment, based on the median number of rural workers, and conducts regression analysis for each group. The results presented in columns (3) and (4) of Table 11 show that the coefficient for the impact of digital rural development in regions with high labor resource endowments is 0.1055, which is significant and positive. This indicates that, following the implementation of digital rural development policies, food security resilience of high labor resource endowments increased by an average of approximately 10.55%, demonstrating a substantial policy effect on enhancing food security resilience. In contrast, for regions with low labor endowments, although a positive coefficient is observed, it did not pass the conventional significance thresholds. This discrepancy can be attributed to the fact that regions with higher labor endowments, benefiting from a relatively higher-quality labor force, are able to more effectively adopt and utilize the new technologies introduced by digital rural development initiatives [63]. This enables them to translate digital support into tangible efficiency gains throughout grain production and distribution. This is achieved through precision farming that safeguards production capacity and smart logistics that stabilize supply, thereby comprehensively enhancing the resilience of food security. Consequently, the “digital divide” is particularly pronounced in regions characterized by low labor resource endowments, where a persistent lack of skilled labor and limited capacity to adopt digital technologies serve as a primary contributor to this disparity. Despite the infrastructure and funding support provided by digital rural development initiatives, the adaptation of the local labor force to digital production tools and models has lagged, hindering the rapid conversion of digital investments into measurable consolidates in food security resilience. As a result, the full policy effects have not yet been fully realized in these regions.

#### 4.6.3. Economic Development Heterogeneity Analysis

Differences in economic development levels also influence the effectiveness of digital rural development in enhancing food security resilience. This study employs the median level of economic development (per capita GDP) as the threshold for grouping and categorizing regions above the median as economically developed. As shown in Columns (1) and (2) of Table 12, the estimated coefficient for the impact of digital rural development on food security resilience in low developed areas is 0.1459, which is significant and positive. This indicates that, following the implementation of digital rural development policies, the food security resilience of low developed areas increased by an average of approximately 14.59%. The selection and positioning of national digital rural pilot zones fundamentally reflect a policy orientation aimed at promoting the digital economy in less developed regions, advancing agricultural digitalization, and consolidating food security. These economically disadvantaged areas have long faced persistent challenges, including entrenched rural issues, a pronounced digital divide, and obstacles to agricultural and rural digital transformation. Digital rural development can propel advances in digital infrastructure, agricultural capital investment, cultivation of digital talent, and the expansion of rural market opportunities. It thereby offers significant developmental prospects for these regions while providing multi-faceted foundational support and momentum for building food security resilience. This consolidation reinforces the security foundation throughout the entire chain from production to distribution.

#### 4.6.4. Local Governance Capacity Heterogeneity Analysis

Local governance capacity serves as the critical conduit for empowering food security resilience through digital rural development. The level of this governance capacity directly influences the extent to which food security resilience fulfills its threefold functions: resistance, recovery, and adaptation. This study divided the research sample into high-governance and low-governance groups based on the median value of the “proportion of villages where the village director and Party secretary positions are held by the same individual (%)”. Regression analysis was then conducted separately for each group. The results in Columns (3) and (4) of Table 12 indicate that the coefficient value for the impact of digital rural development on food security resilience in pilot counties with high local governance capacity is 0.0858, and it is significantly positive. This indicates that, following the implementation of digital rural development policies, the food security resilience of high local governance increased by an average of approximately 8.58%. This demonstrates a significant positive policy effect on enhancing food security resilience in pilot regions. Although a positive trend is observed, pilot areas with low governance capacity have not yet passed the significance test. The reason lies in the fact that regions with high governance capabilities possess well-established governance systems and efficient execution capabilities, enabling them to accurately absorb and transform the policy dividends of digital rural development. Leveraging mature government coordination mechanisms, these regions break down data silos across agriculture, water resources, meteorology, and other sectors to efficiently integrate digital infrastructure, agricultural technology extension, and fiscal support for farming. Simultaneously, initiatives like skill training and service decentralization lower technical barriers for farmers, driving digital empowerment to permeate the entire grain production, distribution, and storage chain, thereby effectively strengthening food security resilience. In contrast, pilot regions with low governance capacity, despite having the foundational conditions for digital development provided by digital village construction, struggle to effectively translate digital investments into enhanced governance efficiency due to their weak governance capabilities. Consequently, they are unable to substantially strengthen food security resilience, resulting in the policy effects needing to be fully realized.

## 5. Conclusions

This study utilizes panel data from major grain-producing counties between 2012 and 2023. Employing the entropy method, it constructs a comprehensive food security resilience index based on three dimensions. This study employs the 2020 “National Digital Rural Pilot Program” policy as a quasi-natural experiment and utilizes a DID approach to investigate the impact of digital rural development on food security resilience in major grain-producing regions, as well as its underlying mechanisms. The main conclusions are as follows: First, the development of digital rural policies has a significant positive impact on the resilience of food security in pilot regions within major grain-producing areas. This conclusion remains valid after undergoing a series of robustness tests [64]. Second, the mechanism analysis reveals that digital rural development exerts its effects by expanding the scale of grain operations in pilot regions and promoting agricultural technological progress [65,66]. Meanwhile, resource allocation efficiency and fiscal transparency exert a positive regulatory effect in enhancing food security resilience through digital rural development [67]. Theoretical models support this view. Finally, compared to non-pilot regions, digital rural development has demonstrated more pronounced policy effects in Southern pilot regions, pilot regions with higher labor resource endowments, pilot regions with lower levels of economic development, and pilot regions with strong local governance capabilities.

The findings of the aforementioned research provide important guidance for the adjustment and promotion of digital rural development policies. In the face of growing uncertainties in the international landscape and rapid domestic urbanization and industrialization, the development of digital villages plays a crucial role in ensuring food security. Firstly, the nation must prioritize securing digital infrastructure resources for major grain-producing regions and deepen the integration of digital technologies with specialty agriculture. Additionally, the nation must develop tailored digital tools suited to the production characteristics of large-scale grain and oilseed cultivation in Northern regions while simultaneously increasing investment in digital infrastructure for Northern rural areas to bridge the “digital divide”, narrowing the gap in digital rural development between Northern and Southern regions, and ensuring digital empowerment covers the entire grain production chain. Secondly, it must actively develop practical skills such as smart agricultural machinery operation, intelligent pest and disease monitoring, and a digital platform based on production to sales matching to address the shortfall in digital literacy. Finally, in light of the reality of global food trade volatility and frequent extreme weather events, leveraging big data to analyze global food market dynamics and conduct real-time risk monitoring will mitigate both internal and external shocks, thereby safeguarding food security and quality. Additionally, strengthening international cooperation on digital agriculture technologies and exchanging best practices drawing on advanced global digital transformation models while adapting them to China’s specific agricultural context will enhance the international competitiveness of China’s food security system.

### Limitations

Currently, there is no universally accepted definition of the “Food Security Resilience Index” within the academic community. This study employs county-level panel data from 2012 to 2023 to construct a comprehensive food security resilience index. Although numerous indicators are incorporated, certain functional variables could not be included in the index system due to data availability constraints. These include the rate of pesticide residues exceeding standards, the nutritional density of food, and the carbon emission intensity of food production. Consequently, the food security resilience index holds potential for further refinement. In addition, our research focuses on the positive effects of digital rural development on food security resilience, with limited analysis of potential negative impacts such as new environmental burdens arising from the large-scale application of digital technologies. Therefore, various factors can be incorporated into future research to deepen the policy research framework for digital rural development.

## Figures and Tables

**Table 1:** Food security resilience indicator system.

Tier-1 Indicator	Tier-2 Indicator	Tier-3 Indicator	Indicator Explanation	Indicator Attributes
Risk Resistance Capacity	Intrinsic Stability	Cultivated Land Area	Total area of cultivated land (hectare)	Positive
		Effective Agricultural Irrigated Area	Area under effective irrigation (hectare)	Positive
	Production–Supply Robustness	Grain Yield per Unit Area	Grain output/sown area of grain crops (kg/hectare)	Positive
		Value-added of the Primary Industry	Gross value added of the primary sector (ten thousand yuan)	Positive
Crisis Recovery Capacity	Sustainability	Chemical Fertilizer Application per Unit Area	Total fertilizer application/sown area of grain crops (kg/hectare)	Negative
		Multiple Cropping Index	Total crop sown area/arable land area (hectare/hectare)	Positive
	Restorability	Rural Electricity Consumption	Total electricity consumption in rural areas (kilowatts/hours)	Positive
		Per Capita Disposable Income in Rural Areas	Average disposable income of rural residents (ten thousand yuan)	Positive
Transformative Capacity	Diverse and Collaboration	Agricultural Fixed Asset Investment	Investment in agricultural fixed assets (ten thousand yuan)	Positive
		Gross Output Value of Agriculture, Forestry, Animal Husbandry, and Fishery	Value added of agriculture, forestry, animal husbandry, and fishery (ten thousand yuan)	Positive
	Production Innovation Level	Agricultural Mechanization Level	Total power of agricultural machinery (kilowatts)	Positive
		Facility Agriculture Area	Area covered by facility agriculture (hectare)	Positive

**Table 2:** Descriptive statistics.

Variable Type	Variable Name	Variable Definition	Mean	Standard Deviation	Minimum Value	Maximum Value
Dependent Variable	FSR	See 3.1.1 for details.	2.2463	0.4078	0.9700	4.9852
Independent Variable	${Policy}_{it}$	See 3.1.2 for details.	0.0104	0.1014	0.0000	1.0000
Mechanism Variables	SGP	Arable Land Area/Persons Engaged in Agriculture, Forestry, Animal Husbandry, and Fishery (hectares/per person)	3.4628	2.7099	1.80 × 10^−9^	5.9984
	ATP	Agricultural Machinery Power/Arable Land Area (kilowatts/hectares)	8.7642	1.5941	0.9976	11.2826
	RAE	Total Grain Output/Arable Land Area (kilogram/hectares)	9.1433	1.4959	0.0110	9.9998
	GFT	China Fiscal Transparency Report	2.7711	0.7633	0.0714	7.0638
Control Variables	LR	Number of Persons Engaged in Agriculture, Forestry, Animal Husbandry, and Fisheries/Rural Workforce (person/person)	0.3441	0.2008	0.0012	0.9984
	AIS	Agricultural Output Value/County-Level GDP (ten thousand yuan/ten thousand yuan)	0.1223	0.1379	0.0001	0.6920
	EDL	Per Capita GDP (ten thousand yuan)	0.2029	0.5120	0.0000	3.5362
	LAM	Area Harvested by Machine/Total Cultivated Land Area (hectares/hectares)	0.3491	0.2994	0.0000	0.9987
	TA	Total Highway Mileage/Administrative Area (kilometers/square kilometer)	0.0689	0.0724	0.0000	0.3627
	NAE	(Secondary Industry Value Added + Tertiary Industry Value Added)/Regional Gross Domestic Product) (ten thousand yuan/ten thousand yuan)	0.5823	0.1181	0.0014	0.7302

**Table 3:** Benchmark regression results.

Variable	FSR			
	(1)	(2)	(3)	(4)
${Policy}_{it}$	0.1440 ***	0.0876 *	0.1330 ***	0.0898 *
	(0.0368)	(0.0483)	(0.0351)	(0.0481)
LR			0.0364 **	0.0476 **
			(0.0178)	(0.0200)
AIS			−0.0985 ***	−0.0319
			(0.0281)	(0.0509)
EDL			−0.1558 ***	−0.0171
			(0.0070)	(0.0105)
NAE			−0.2427 ***	−0.1085
			(0.0304)	(0.1498)
TA			−1.0639 ***	−0.0650
			(0.0536)	(0.0976)
LAM			0.0560 ***	−0.0066
			(0.0120)	(0.0207)
County-Fixed Effects	No	Yes	No	Yes
Year-Fixed Effects	No	Yes	No	Yes
Constant Term	2.2448 ***	2.2454 ***	2.4712 ***	2.3063 ***
	(0.0038)	(0.0005)	(0.0205)	(0.0888)
N	11,834	11,834	11,834	11,834
*R* ^2^	0.001	0.584	0.093	0.584

Note: ***, **, and * denote significance levels of 1%, 5%, and 10%, respectively. The values in parentheses represent the robust standard errors of the variable coefficients for county-level clustering; the same applies below.

**Table 4:** Temporal placebo test results.

Variable	One Year in Advance	Two Years in Advance	Three Years in Advance	Four Years in Advance
	(1)	(2)	(3)	(4)
${Policy}_{it}$	0.0558	0.0517	0.0471	0.0527
	(0.0517)	(0.0563)	(0.0503)	(0.0478)
LR	0.0478 **	0.0479 **	0.0479 **	0.0480 **
	(0.0200)	(0.0200)	(0.0200)	(0.0200)
AIS	−0.0318	−0.0315	−0.0315	−0.0316
	(0.0509)	(0.0509)	(0.0510)	(0.0510)
EDL	−0.0170	−0.0170	−0.0169	−0.0168
	(0.0105)	(0.0105)	(0.0105)	(0.0105)
NAE	−0.1075	−0.1071	−0.1068	−0.1067
	(0.1497)	(0.1496)	(0.1496)	(0.1496)
TA	−0.0657	−0.0656	−0.0657	−0.0657
	(0.0975)	(0.0975)	(0.0975)	(0.0975)
LAM	−0.0060	−0.0059	−0.0058	−0.0059
	(0.0207)	(0.0207)	(0.0207)	(0.0207)
County-Fixed Effects	Yes	Yes	Yes	Yes
Year-Fixed Effects	Yes	Yes	Yes	Yes
Constant Term	2.3057 ***	2.3053 ***	2.3050 ***	2.3047 ***
	(0.0887)	(0.0887)	(0.0887)	(0.0887)
N	11,834	11,834	11,834	11,834
*R* ^2^	0.584	0.584	0.584	0.584

Note: *** and ** denote significance levels of 1% and 5%, respectively. The values in parentheses represent the robust standard errors of the variable coefficients for county-level clustering; the same applies below.

**Table 5:** Results of propensity score matching and entropy balancing tests.

Variable	Baseline Regression	Propensity Score Matching	Entropy Balance Test
	(1)	(2)	(3)
${Policy}_{it}$	0.0898 *	0.0896 *	0.0838 *
	(0.0481)	(0.0482)	(0.0483)
LR	0.0476 **	0.0504 **	0.0352
	(0.0200)	(0.0200)	(0.0668)
AIS	−0.0319	−0.0383	0.0507
	(0.0509)	(0.0513)	(0.0713)
EDL	−0.0171	−0.0176 *	−0.0113
	(0.0105)	(0.0105)	(0.0184)
NAE	−0.1085	−0.0881	−0.0323
	(0.1498)	(0.1406)	(0.1822)
TA	−0.0650	−0.0849	−0.1962
	(0.0976)	(0.0982)	(0.4009)
LAM	−0.0066	−0.0067	0.0077
	0.0898 *	(0.0207)	(0.0491)
County-Fixed Effects	Yes	Yes	Yes
Year-Fixed Effects	Yes	Yes	Yes
Constant Term	2.3063 ***	2.2948 ***	2.3061 ***
	(0.0888)	(0.0837)	(0.1149)
N	11,834	11,796	11,834
*R* ^2^	0.584	0.584	0.641

Note: ***, **, and * denote significance levels of 1%, 5%, and 10%, respectively. The values in parentheses represent the robust standard errors of the variable coefficients for county-level clustering; the same applies below.

**Table 6:** Regression results controlling for other policy interferences.

Variable	National Safe Agricultural Machinery Demonstration County	National Modern Agricultural Demonstration Zone	National Model County for Rural Revitalization	National Pilot County/City for Agricultural Product Quality and Safety
	(1)	(2)	(3)	(4)
${Policy}_{it}$	0.0900 *	0.0897 *	0.0909 *	0.0903 *
	(0.0482)	(0.0482)	(0.0482)	(0.0482)
LR	0.0473 **	0.0478 **	0.0477 **	0.0472 **
	(0.0200)	(0.0200)	(0.0200)	(0.0200)
AIS	−0.0329	−0.0317	−0.0316	−0.0312
	(0.0509)	(0.0510)	(0.0507)	(0.0509)
EDL	−0.0173 *	−0.0172	−0.0170	−0.0172
	(0.0105)	(0.0105)	(0.0105)	(0.0105)
NAE	−0.1079	−0.1085	−0.1086	−0.1083
	(0.1499)	(0.1498)	(0.1497)	(0.1498)
TA	−0.0640	−0.0673	−0.0623	−0.0647
	(0.0976)	(0.0978)	(0.0978)	(0.0974)
LAM	−0.0067	−0.0068	−0.0069	−0.0067
	(0.0207)	(0.0207)	(0.0207)	(0.0207)
County-Fixed Effect	Yes	Yes	Yes	Yes
Year-Fixed Effects	Yes	Yes	Yes	Yes
Constant Term	2.3059 ***	2.3068 ***	2.3063 ***	2.3059 ***
	(0.0888)	(0.0888)	(0.0887)	(0.0888)
N	11,834	11,834	11,834	11,834
*R* ^2^	0.584	0.584	0.584	0.584

Note: ***, **, and * denote significance levels of 1%, 5%, and 10%, respectively. The values in parentheses represent the robust standard errors of the variable coefficients for county-level clustering; the same applies below.

**Table 7:** Regression results from other robustness tests.

Variable	Year–Province-Fixed Effects Model	Excluding County-Level Cities	One-Period Lag of Policy
	(1)	(2)	(3)
${Policy}_{it}$	0.0835 *	0.1128 *	0.0295 **
	(0.0491)	(0.0607)	(0.0116)
LR	0.0454 **	0.0598 ***	0.0150 **
	(0.0203)	(0.0220)	(0.0076)
AIS	−0.0297	−0.0575	0.0104
	(0.0503)	(0.0620)	(0.0141)
EDL	−0.0111	−0.0160	−0.0053
	(0.0107)	(0.0122)	(0.0034)
NAE	−0.1395	−0.0046	0.0630 ***
	(0.1547)	(0.1572)	(0.0156)
TA	−0.1234	−0.0162	−0.0260
	(0.1123)	(0.1082)	(0.0252)
LAM	−0.0122	−0.0016	0.0290 ***
	(0.0208)	0.1128 *	(0.0071)
County-Fixed Effects	Yes	Yes	Yes
Year-Fixed Effects	Yes	Yes	Yes
Year–Province-Fixed Effects	Yes	No	No
Constant Term	2.3297 ***	2.2334 ***	0.3119 ***
	(0.0913)	(0.0929)	(0.0097)
N	11,834	9520	11,834
*R* ^2^	0.590	0.584	0.712

Note: ***, **, and * denote significance levels of 1%, 5%, and 10%, respectively. The values in parentheses represent the robust standard errors of the variable coefficients for county-level clustering; the same applies below.

**Table 8:** Mediated effect analysis results.

Variable	Baseline Regression	SGP	ATP
	(1)	(2)	(3)
${Policy}_{it}$	0.0898 *	0.2461 **	0.2532 *
	(0.0481)	(0.1153)	(0.1394)
LR	0.0476 **	0.0588	0.0174
	(0.0200)	(0.0488)	(0.0686)
AIS	−0.0319	−0.0350	0.3514 **
	(0.0509)	(0.1379)	(0.1462)
EDL	−0.0171	−0.8676 ***	0.0595
	(0.0105)	(0.0804)	(0.0473)
NAE	−0.1085	−0.3687 **	−0.2646
	(0.1498)	(0.1827)	(0.2382)
TA	−0.0650	−0.0670	−0.7694
	(0.0976)	(0.3214)	(0.5289)
LAM	−0.0066	−0.0221	−0.0141
	(0.0207)	(0.0369)	(0.0597)
County-Fixed Effects	Yes	Yes	Yes
Year-Fixed Effects	Yes	Yes	Yes
Constant Term	2.3063 ***	3.8473 ***	8.9127 ***
	(0.0888)	(0.1159)	(0.1521)
N	11,834	11,834	11,834
*R* ^2^	0.584	0.938	0.684

Note: ***, **, and * denote significance levels of 1%, 5%, and 10%, respectively. The values in parentheses represent the robust standard errors of the variable coefficients for county-level clustering; the same applies below.

**Table 9:** Results of the mediation effect test based on the Bootstrap method.

Mediator Variables	Effect Type	Effect	Bootstrap Std. Err.	Confidence Interval (P)	Confidence Interval (BC)
SGP	Indirect effects	0.00011	0.00006	[0.000014, 0.00025]	[0.00003, 0.00030]
ATP	Indirect effects	0.00034	0.00013	[0.00012, 0.00063]	[0.00013, 0.00065]

**Table 10:** Moderation effect analysis results.

Variable	Baseline Regression	RAE	GFT
	(1)	(2)	(3)
${Policy}_{it}$	0.0898 *	0.0722 *	0.0716 *
	(0.0481)	(0.0436)	(0.0401)
RAE* ${Policy}_{it}$		0.0613 ***	
		(0.0111)	
GFT* ${Policy}_{it}$			0.1274 **
			(0.0559)
LR	0.0476 **	0.0454 **	0.0484 **
	(0.0200)	(0.0200)	(0.0199)
AIS	−0.0319	−0.0318	−0.0309
	(0.0509)	(0.0508)	(0.0509)
EDL	−0.0171	−0.0138	−0.0150
	(0.0105)	(0.0113)	(0.0115)
NAE	−0.1085	−0.1110	−0.1064
	(0.1498)	(0.1499)	(0.1500)
TA	−0.0650	−0.0679	−0.0664
	(0.0976)	(0.0977)	(0.0974)
LAM	−0.0066	−0.0059	−0.0077
	(0.0207)	(0.0207)	(0.0207)
RAE		0.0039	
		(0.0042)	
GFT			−0.0095
			(0.0244)
County-Fixed Effects	Yes	Yes	Yes
Year-Fixed Effects	Yes	Yes	Yes
Constant Term	2.3063 ***	2.2721 ***	2.3311 ***
	(0.0888)	(0.0957)	(0.1125)
N	11,834	11,834	11,834
*R* ^2^	0.584	0.585	0.585

Note: ***, **, and * denote significance levels of 1%, 5%, and 10%, respectively. The values in parentheses represent the robust standard errors of the variable coefficients for county-level clustering; the same applies below.

**Table 11:** Results of heterogeneity analysis.

Variable	Regional Heterogeneity Analysis		Analysis of Heterogeneity in Agricultural Labor Force Endowments	
	South	North	High	Low
	(1)	(2)	(3)	(4)
${Policy}_{it}$	0.2010 **	0.0011	0.1055 **	0.0829
	(0.0939)	(0.0421)	(0.0504)	(0.0863)
LR	0.0471	0.0557 **	0.0250	0.0220
	(0.0330)	(0.0241)	(0.0398)	(0.0275)
AIS	−0.0553	−0.0355	0.0943	−0.0970
	(0.0991)	(0.0518)	(0.0841)	(0.0683)
EDL	−0.0290 *	−0.0129	−0.0250 **	−0.0128
	(0.0153)	(0.0137)	(0.0112)	(0.0191)
NAE	0.5756 **	0.1440	−0.0897	−0.1343
	(0.2348)	(0.1742)	(0.2187)	(0.1757)
TA	0.4769 *	−0.1685	−0.1284	−0.0130
	(0.2614)	(0.1045)	(0.1755)	(0.1275)
LAM	0.0029	−0.0172	0.0186	−0.0332
	(0.0314)	(0.0273)	(0.0281)	(0.0321)
County-Fixed Effects	Yes	Yes	Yes	Yes
Year-Fixed Effects	Yes	Yes	Yes	Yes
Constant Term	2.5875 ***	2.1516 ***	2.2789 ***	2.3454 ***
	(0.1437)	(0.1000)	(0.1309)	(0.1053)
N	5027	6807	5812	5875
*R* ^2^	0.573	0.594	0.594	0.607

Note: ***, **, and * denote significance levels of 1%, 5%, and 10%, respectively. The values in parentheses represent the robust standard errors of the variable coefficients for county-level clustering; the same applies below.

**Table 12:** Results of heterogeneity analysis.

Variable	Economic Development Heterogeneity Analysis		Local Governance Capacity Heterogeneity Analysis	
	High	Low	High	Low
	(1)	(2)	(3)	(4)
${Policy}_{it}$	0.0761	0.1459 **	0.0858 *	0.1197
	(0.0730)	(0.0736)	(0.0439)	(0.0997)
LR	0.0014	0.0840 ***	0.0477 *	0.0683 **
	(0.0260)	(0.0322)	(0.0273)	(0.0302)
AIS	0.1780	−0.0998 *	−0.0382	−0.0319
	(0.1213)	(0.0544)	(0.0588)	(0.0840)
EDL	−0.0279 **	−0.0200	−0.0467 ***	0.0027
	(0.0124)	(0.0202)	(0.0171)	(0.0134)
NAE	−0.2100	−0.0407	−0.0454	−0.2361
	(0.2287)	(0.2011)	(0.1899)	(0.2863)
TA	−0.2131	−0.0152	−0.3554 ***	0.5387 ***
	(0.2544)	(0.1189)	(0.1187)	(0.1645)
LAM	−0.0227	0.0110	−0.0170	0.0135
	(0.0257)	(0.0356)	(0.0271)	(0.0275)
County-Fixed Effects	Yes	Yes	Yes	Yes
Year-Fixed Effects	Yes	Yes	Yes	Yes
Constant Term	2.3652 ***	2.2715 ***	2.3523 ***	2.2674 ***
	(0.1374)	(0.1164)	(0.1115)	(0.1745)
N	6023	5756	5915	5710
*R* ^2^	0.577	0.598	0.558	0.616

Note: ***, **, and * denote significance levels of 1%, 5%, and 10%, respectively. The values in parentheses represent the robust standard errors of the variable coefficients for county-level clustering; the same applies below.

## Data Availability

The original contributions presented in the study are included in the article; further inquiries can be directed to the corresponding author.

## Appendix A

To interpret the indicators of the food security resilience system more objectively, the entropy method was employed to refine and adjust the weights of each indicator. First, data standardization was performed, as follows:

Eliminate the effects of dimension and magnitude and standardize positive and negative indicator data. The standardization method is as follows:

(A1) $$s=\sum_{i=1}^{m}\sum_{j=1}^{n}\;(X_{ij}.W_{i})$$

Positive indicators indicate that higher values are better, while negative indicators indicate that lower values are better.

In Formula (A1), $W_{i}$ represents the weight of the indicator, and $X_{ij}$ is the standardized value of the j

-th indicator for the i-th county, where

(A2) $$Positive\;indicators:X_{ij}=\frac{X_{ij}-minX_{ij}}{maxX_{ij}-minX_{ij}}$$

In Equations (A2) and (A3), $X_{ij}$ denotes the value of the j-th indicator for the i-th county ( i = 1, 2, 3, …, m; j = 1, 2, 3, …, n). Next, indicator weights are constructed as follows: based on data standardization, calculate the proportion $P_{ij}$ of the jth evaluation indicator for the i-th county, the information entropy $E_{ij}$ of the jth indicator, the weight $W_{j}$ of the j-th indicator, and the food security resilience $R_{i}$ of the i-th county.

Calculate the proportion of the i-th sample under the j-th indicator as follows:

(A3) $$P_{ij}=X_{ij}/\sum_{i=1}^{m}X_{ij}$$

Calculate the entropy value for the  j-th indicator as follows:

(A4) $$E_{j}=-k\sum_{i=1}^{m}P_{ij}\times \ln P_{ij},k=\frac{1}{\ln m}$$

Calculate information entropy redundancy as follows:

(A5) $$d_{j}=1-E_{j},\;j=1,2,\ldots ,m$$

Calculate the weight of each indicator as follows:

(A6) $$W_{i}=\frac{1-E_{ij}}{n-\sum_{j=1}^{n}E_{ij}}$$

Finally, calculate the comprehensive food security resilience index as follows:

(A7) $$R_{i}=\sum_{j=1}^{n}W_{j}X_{ij}$$

where ${\;X}_{ij}$ represents the standardized data.

The value of information entropy redundancy $d_{j}$ influences the magnitude of indicator weight $W_{i}$. Greater redundancy contributes more significantly to the evaluation of food security resilience indicators, resulting in a higher indicator weight. A higher composite score $R_{i}$ indicates better food security resilience in a region.
